# Supplementary material for: Safety and Immunogenicity of Combined DNA-Polyethylenimine and Oral Bacterial Idiotypic Vaccine for Patients with B-Cell Non-Hodgkin Lymphoma: A Pilot Study
Source: Cancers (Basel). 2022 Jul 6;14(14):3298. doi: 10.3390/cancers14143298 (PMC9320637; doi:10.3390/cancers14143298)
Supplement: Supplementary file 1 [file cancers-14-03298-s001.zip › cancers-1767855-supplementary.pdf]

Supplementary Materials: Safety and Immunogenicity of Combined DNA-Polyethylenimine and Oral Bacterial Idiotypic Vaccine for Patients with B-Cell Non-Hodgkin Lymphoma: A Pilot Study

Alexander Meleshko, Nadzeya Piatrouskaya, Katsiaryna Vashkevich, Dzmitry Lutskovich, Chuan Wang, Dmitri Dormeshkin, Natalia Savelyeva and Mikalai Katsin

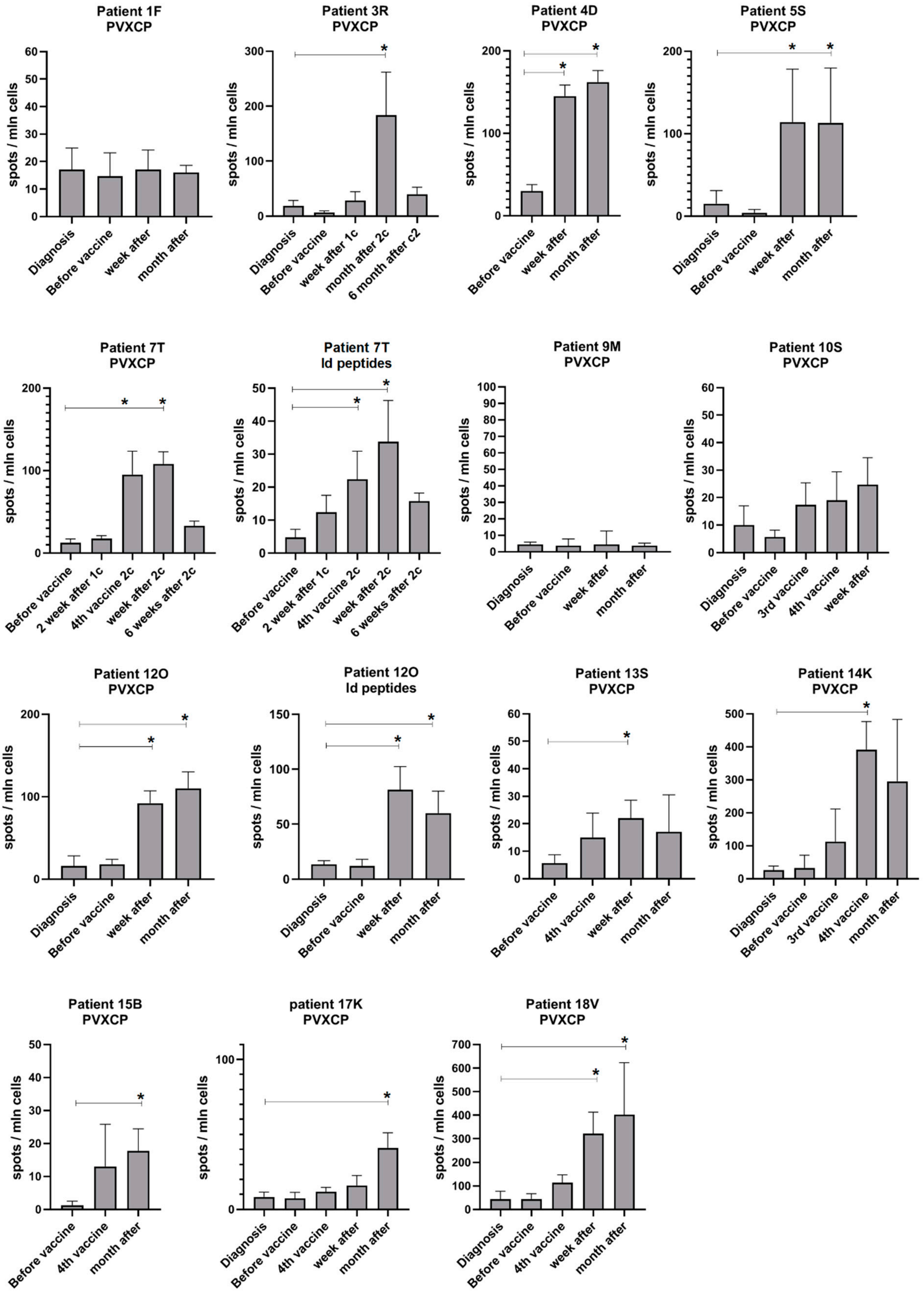

Figure S1. ELISpot test for all patients. 1c—first vaccine course, 2c—second vaccine course (indicated only for patients who have had two courses of vaccination). For some patients, material was available at additional points (on the day of the 3rd or 4th vaccine), these points are also presented in the graph. A significant difference ($p < 0.05$) in the number of spots between time points is indicated by the asterisk (Student's *t*-test).

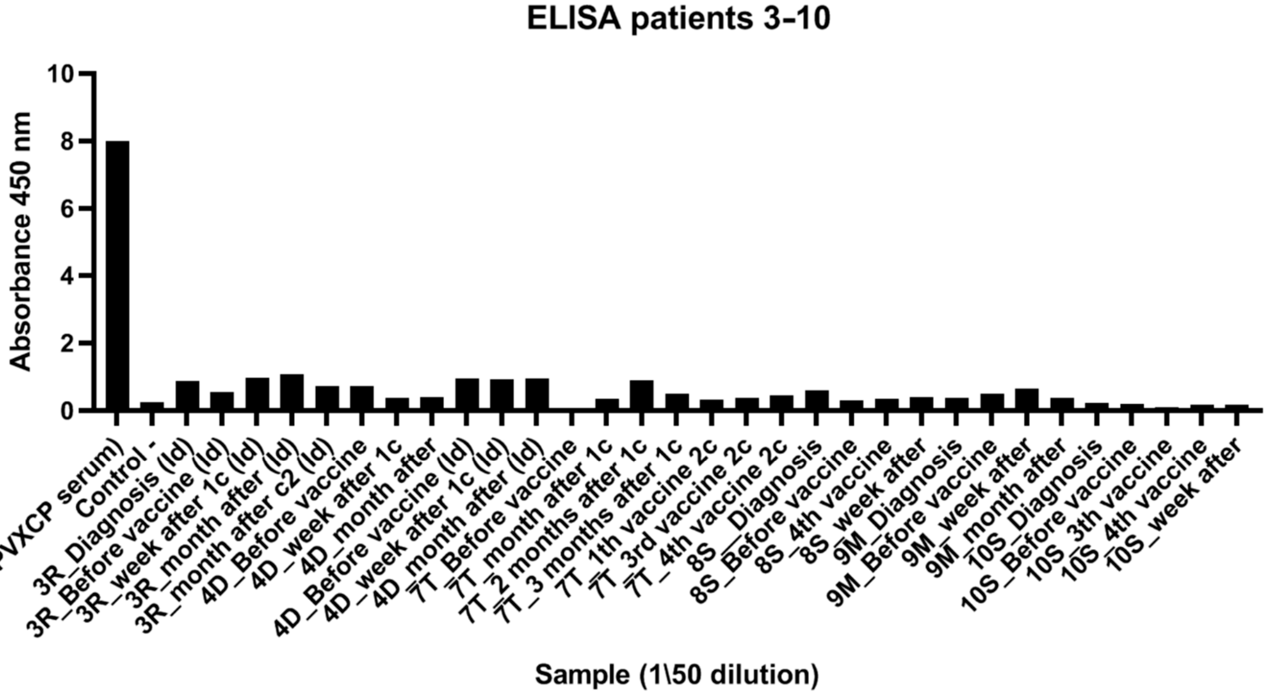

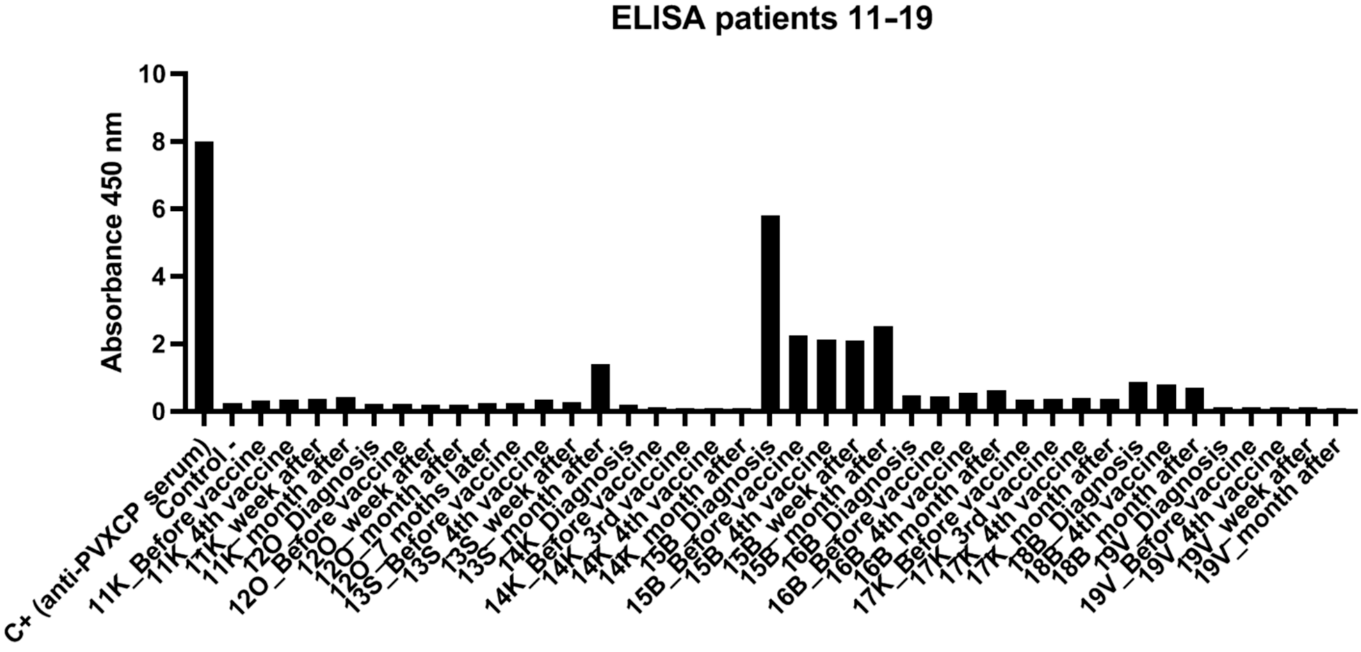

Figure S2. ELISA test for all patients and controls. Id—antigen is the patient's idiotype protein, other samples tested against PVXCP protein. 1c—first vaccine course, 2c—second vaccine course (indicated only for patients who have had two courses of vaccination). Positive control—mouse immune serum, negative control—medium.

Table S1. Sequences of variable regions (idiotypes) of patients' Ig genes included in the study, and the degree of germline homology.

Patient	Idiotype	Sequence	VH/VL Germline
1F	IgM,D	ATGGACTGGACCTGGAGGTTCCTCTTTGTGGTGGCAGCAGCTACAGGTGTCCAGTCCCAGGTGCAGCTGGTGCAGTCTGGGGCTGAGGTGAAGAAGCCTGGGTCCTCGGTGAAGGTCTCCTGCAAGGCTTCTGGAGGCACCTTCAGCAGCTATGCTATCAGCTGGGTGCGACAGGCCCCTGGACAAGGGCTTGAGTGGATGGGAGGGATCATCCCTATCTTTGGTACAGCAAACTACGCACAGAAGTTCCAGGGCAGAGTCACGATTACCGCGGACGAATCCACGAGCACAGCCTACATGGAGCTGAGCAGCCTGAGATCTGAGGACACGGCCGTGTATTACTGTGCGAGAGTCCAATCGGCGATTTTTGGAGTGGTTATTCTTGGGCCTTACTACTACTACATGGACGTCTGGGGCAAAGGGACCACGGTCACCGTCTCCTCA	100%
1F	IgK	GACATCCAGATGACCCAGTCTCCATCTTCCGTGTCTGCATCTGTAGGAGACAGAGTCACCATCACTTGTCGGGCGAGTCAGGGTATTAGCAGCTGGTTAGCCTGGTATCAGCAGAAACCAGGGAAAGCCCCTAAGCTCCTGATCTATGCTGCATCCAGTTTGCAAAGTGGGGTCCCATCAAGGTTCAGCGGCAGTGGATCTGGGACAGATTTCACTCTCACCATCAGCAGCCTGCAGCCTGAAGATTTTGCAACTTACTATTGTCAACAGGCTAACAGTTCCCGTGACCCTGGGACCAAAGTGGATATCAAAC	100%
2K	IgD	ATGAAACACCTGTGGTTCTTCCTCCTCCTGGTGGCAGCTCCCAGATGGGTCCTGTCCCAGGTGCAGCTGCAGGAGTCGGGCCCAGGACTGGTGAAGCCTTCGGAGACCCTGTCCCTCACCTGCACTGTCTCTGGTGGCTCCGTCAGCAGTGGTAGTTACTACTGGAGCTGGATCCGGCAGCCCCCAGGGAAGGGACTGGAGTGGATTGGGTATATCTATTACAGTGGGACCACCAACTACAACCCCTCCCTCAAGAGTCGAGTCACCATATCAGTAGACACGTCCAAGAACCAGTTCTCCCTGAAGCTGAGCTCTGTGACCGCTGCGGACACGGCCGTGTATTACTGTGCGAGACCGTCCTACCCGAACGACGAGGATGCTTTTGATATCTGGGGCCAAGGGACAATGGTCACCGTCTCTTCAG	99.6%
2K	IgK	TCCTATGTGCTGACTCAGCCACCCTCAGTGTCAGTGGCCCCAGGAAAGACGGCCAGGATTACCTGTGGGGGAAACAACATTGGAAGTAAAAGTGTGCACTGGTACCAGCAGAAGCCAGGCCAGGCCCCTGTGCTGGTCATCTATTATGATAGCGACCGGCCCTCAGGGATCCCTGAGCGATTCTCTGGCTCCAACTCTGGGAACACGGCCACCCTGACCATCAGCAGGGTCGAAGCCGGGGATGAGGCCGACTATTACTGTCAGGTGTGGGATAGTAGTAGTGATCTTCCTTGGGTGTTCGGCGGAGGGACCAAGCTGACCGTCCTA	100%
3R	IgM,D	ATGAAACATCTGTGGTTCTTCCTTCTCCTGGTGGCAGCTCCCAGATGGGTCCTGTCCCAGGTGCAGCTGCAGGAGTCGGGCCCAGGACTGGTGGAGCCTTCGGAGACCCTGTCCCTCACTTGCACTGTTTCTGGTGGCTCCATGAGTGGATACTACTGGAGCTGGATGCGGCAGTCCCCAGGGAAGGGACTGGAGTGGATTGGATATATCTATTACAGTGGGACCACCGACTACAACCCCTCCCTCAAGAGTCGAGTCACCATATCAGTGGACACGTCCAAGAATCAGTTCTCCCTGAAGTTGAGCTCTGTGACCGCTGCGGACACGGCCGTCTATTACTGTGCGAGAGACGCCCCTATGAAGTATAGTAGTTTGTCAGGGTACTACTATTACGGCATGGACGTCTGGGGCCAAGGGACCGCGGTCACCGTCTCCTCAG	94.74%
3R	IgK	GACATCCAGATGACCCAGTCTCCATCCTCCCTGTCTGCATCTGTAGGAGACAGAGTCACCATCACTTGCCGGGCAAGTCAGAGCATTAGCAGTTATTTAAATTGGTATCAGCAAATACGAGGGAAAGCCCCTAAGCTCCTGACTTATGCTGCTACCACTTTGCAAAGTGGGGTCCCATCAAGGTTCAGTGGCAGTGGATCTGGGACAGATTTCACTCTCACCATCAGCAGTCTACAACCCGAAGACCTTGCAACTTACTATTGTCAACAGACTTACAGTACCCCGTGGACGTTCGGCCAAGGGACCAGGGTGGAAATCAAAC	94.62%
4D	IgM,D	ATGGAGTTTGGGCTGAGCTGGCTTTTTCTTGTGGCTAAAATAAAAGGTGTCCAGTGTGAGGTGCAGCTGTTGGAGTCTGGGGGAGGCTTGGTACAGCCTGGGGGGTCCCTGAGACTCTCCTGTGCAGCCTCTGGATTCACCTTTAGCAGCTATGCCATGACCTGGGTCCGCCAGGCCCCAGGGAAGGGGCTGGAGTGGGTCTCAGCTATAAGTGGTAGTGGTGGTAGCACATACTACGCAGACTCCGTGAGGGGCCGGTTCACCATCTCCAGAGACAATTCCAAAAACACGCTTTATCTGCAAATGACCAGCCTGAGAGCCGAGGACACGGCCGTATATTACTGTGCGAAGATCCCGACGGTGACTAACTACTACTTGGACTCCTGGGGCCAGGGGACCCTGGTCACCGTCTCCTCAG	97.57%
4D	IgK	GACATCCAGATGACCCAGTCTCCTTCCACCCTGTCTGCATCTGTAGGAGACAGAGTCACCATCACTTGCCGGGCCAGTCAGAGTATTAGTAGCTGGTTGGCCTGGTATCAGCAGAAACCAGGGAAAGCCCCTGAGCTCCTGATTTATAGGGCGTCTACTTTACAAAGTGGGGTCCCATCACGTTTCAGCGGCAGTGGATCTGGGACAGAATTCACTCTCACCATCAGCTGCCTGCAGCCTGATGATTTTGCAACTTATTACTGCCAACAGTATAATAGTTATTTACTCACTTTCGGCGGAGGGACCAAGGTGGAGATCAAAC	97.13%
5S	IgG	ATGGAGTTTGGGCTGAGCTGGGTTTTCCTTGTTGCTATTTTAAAAGGTGTCCAGTGTGAGGTGCAGCTGGTGGAGTCTGGGGGAGGCTTGGTCCAGCCTGGGGGGTCCCTGAGACTCTCCTGTGCAGCCTCTGGATTCTCCTTTGGTGGCTATTGGATGACCTGGGTCCGCCAGGCTCCAGGGAAGGGGCTGGAGTGGGTGGCCAACATAAACCAAGATGGAAGTGACAAATACTATCTGGACTCTGTGGAGGGCCGATTCACCATCTCCAGAGACAGCGCCAAGAACTCCCTGTATCTGCAAATGAACAGCCTCAGAGCCGAGGACACGGCCATATATTACTGTGCGAGAGCGCAAGATTTTGAGACTATAGCAGCAGCTGTCTGGGGCCAGGGGACCCAGGTCACCGTCTCCGCAC	95.49%
5S	IgL	CAGCCGCCCTCGGTGTCAGTGTCCCCAGGACAAACGGCCAGGATCACCTGCTCTGGAGATGCATTGCCAAAAAAATTTGCTTATTGGTACCAGCAGAGGTCAGGCCAGGCCCCTGTGCTGGTCATCTATGAGGACAGTAAGCGACCCTCCGGGATCCCTGAGAGATTCTCTGGCTCCAGCTCAGGGACAATGGCCACCTTGACTATCAGTGGGGCCCACGTGGAGGATGAAGCTGACTACTATTGTTACTCAACAGACAGCAGTGGTAATCATTGGGTGTTCGGCGGAGGGACCAAGCTGACCGTCCTA	97.35%
6L	IgM,D	ATGGACTGGACCTGGAGGATCCTCTGCTTGCTGGCTGTAGCTCCAGGTGCTCACTCCCAGGTGCAGCTGGTGCAGTCTGGGGCTGAGGTGAAGAAGCCTGGGGCCTCAGTGAAGGTTTCCTGCAAGGCATCTGGATACACCTTCACCAGCTACTATATGCACTGGGTGCGACAGGCCCCTGGACAAGGGCTTGAGTGGATGGGAATAATCAACCCTAGTGGTGGTAGCACAAGCTACGCACAGAAGTTCCAGGGCAGAGTCACCATGACCAGGGACACGTCCACGAGCACAGTCTACATGGAGCTGAGCAGCCTGAGATCTGAGGACACGGCCGTGTATTACTGTGCGAGAGAGTATCCCTTAGTAGTACCAGCTGCTATATACTACTACTACTACGGTATGGACGTCTGGGGCCAAGGGACCACGGTCACCGTCTCCTCAG	100%
6L	IgK	TCATCACCACGATATTGTGATGACCCAGACTCCACTCTCCCTGCCCGTCACCCCTGGAGAGCCGGCCTCCATCTCCTGCAGGTCTAGTCAGAGCCTCCTGCATAGTAATGGATACAACTATTTGGATTGGTACCTGCAGAAGCCAGGGCAGTCTCCACAGCTCCTGATCTATTTGGGTTCTAATCGGGCCTCCGGGGTCCCTGACAGGTTCAGTGGCAGTGGATCAGGCACAGATTTTACACTGAAAATCAGCAGAGTGGAGGCTGAGGATGTTGGGGTTTATTACTGCATGCAAGCTCTACAAACTCCTCCATTCACTTTCGGCCCTGGGACCAAAGTGGATATCAAA	99.33%
7T	IgM	ATGGACTGGACCTGGAGGTTCCTCTTTGTGGTGGCAGCAGCTACAGGTGTCCAGTCCCAGGTCCAGCTGGTGCAATCTGGGGCTGAGTTGAAGAAGCCTGGGTCCTCGGTGAAGGTCTCCTGCAAGTCTTCTGGAGACACTTTCAACAAGTATGCTTTAAGTTGGCTGCGACAGGCCCCTGGACAAGGGCCTGAGTGGATGGGAAGCATCATCCCTCTCCTTGGTACTGCAAATTACGCACAGAAATTCCAGGGCAGGGTCACGATTACCGCGGACAAATCCACGAGTACAACCTATTTGGAATTGAAGAGCCTGCGATTTGAAGACACGGCCGTATATTACTGTGCGAGAGGGTGGTCAGGGACTGGGACGGCCTCCGGATTCAACTACTGGGGCCAGGGAACCCTGGTCAGCGTCTCCTCA	89.24%
7T	IgK	GAAATTGTGTTGACGCAGTCTCCAGGCACCCTGTCTTTGTCTCCAGGGGAAAGGGCCACTCTCTCCTGCAGGGCCAGTCAGAGTGTTAGTGCCAACTCTTTAGCCTGGTACCAGCAAAAACCTGGCCGGCCTCCCAGGCTCCTCATCTATGGTGCATTCAGCAGGGCCACTGGCATCCCAGACAGGTTCAGTGCCAGTGGGTCTGGGACAGACTTCACTCTCACCATCAGCAGACTGGAGCCTGAAGATTTTGTAGTGTATTACTGTCAGCACTATGGTAGTTCACCTCGGACGTTCGGCCAAGGGACCAAGGTGGAAATCAAAC	94.33%

8S	IgM	ATGGAGTTTGGGCTGAGCTGGCTTTTTCTTGTGGCTAAAATAAAAGGTGTCCAGTGTGAGGTGCAGATGTTGGAGTCTGGGGGAG<br>GCTTGATACAGCCGGGGGGTCCCTGAGACTCTCCTGTGAAGCCTCTGGATTCACCTTTAGCAGCTATGCCATGAGTTGGGTCCG<br>CCAGGCTCCAGGGCAGGGGCTGGAGTGGGTCTCCGGTATCAGCAGTAGTGGTGGGACCACATACTACACAGACTCCGTGAAGGGC<br>CGGTTCACCATCTCCAGAGACAATACCAAGAACACTTTATATCTGGAAATGAAGAGGCTGAGAGTCGAGGACACGGCCTTATATT<br>ACTGTTCGAAAGATCAGGCGCAACAACTCTTACTAGTTTCCCTTAGTTACTGGTATTTCGATCTCTGGGGCCGCGGCACCCTGGT<br>CACTGTGTCCTCAG	92.01%
8S	IgK	GCCATCCGGATGACCCAGTCTCCATCCTCATTCTCTGCGTCTTCAGGAGACAGAGTCAACATCACTTGTCGGGCGAGTCAGGGTA<br>TTAGCAGTTACTTGGCCTGGTATCAGCAAAAATTAGGGGAGGCCCCTAAACTCCTGATCTATTCTGCATCCACTTTGCAAAGTGG<br>GGTCCCATCAAGGTTCAGCGGCAGTGGCTCTGGGACAGATTTCACTCTCACCATCAGCTGCCTGCAGTCTGAAGACTTTGCAACT<br>TATTACTGTCAACAGTATTATAGTTACCCACTCACTTTCGGCGGAGGGACCAACATTGACATCAAAC	95.34%
9M	IgM	ATGGACTGGACCTGGAGGTTCCTCTTTGTGGTGGCAGCAGCTACAGGTGTCCAGTCCCAGGTGCAGCTGGTGCAGTCTGGGGCTG<br>AGGTGAAGAAGCCTGGGTCCTCGGTGAAGGTCTCCTGCAAGGCTTCTGGAGGCACCTTCAGCAGCTATGCTATCAGCTGGGTGCG<br>ACAGGCCCCTGGACAAGGGCTTGAGTGGATGGGAGGGATCATCCCTATCTTTGGTACAGCAAACTACGCACAGAAGTTCCAGGGC<br>AGAGTCACGATTACCGCGGACGAATCCACGAGCACAGCCTACATGGAGCTGAGCAGCCTGAGATCTGAGGACACGGCCGTGTATT<br>ACTGTGCGAGCGTCGAGGTACCTGGAACTACGTTCACGCTTGACTACTGGGGCCAGGGAACCCTGGTCACCGTCTCCTCAG	100%
9M	IgL	CAGTCTGTGCTGACTCAGCCACCCTCAGCGTCTGGGACCCCCGGGCAGAGGGTCACCATCTCTTGTTCTGGAAGCAGCTCCAACA<br>TCGGAAGTAATTATGTATACTGGTACCAGCAGCTCCCAGGAACGGCCCCCAAACTCCTCATCTATAGGAATAATCAGCGGCCCTC<br>AGGGGTCCCTGACCGATTCTCTGGCTCCAAGTCTGGCACCTCAGCCTCCCTGGCCATCAGTGGGCTCCGGTCCGAGGATGAGGCT<br>GATTATTACTGTGCAGCATGGGATGACAGCCTGAGTGGTCGGGTGTTCGGCGGAGGGACCAAGCTGACCGTCCTAG	98.92%
10S	IgM,D	ATGAAACACCTGTGGTTCTTCCTCCTCCTGGTGGCAGCTCCCAGATGGGTCCTGTCCCAGGTGCAGCTACAGCAGTGGGGCGCAG<br>GACTGTTGAAGCCTTCGGAGACCCTGTCCCTCACCTGCGCTGTCTATGGTGGGTCCTTCAGCGGTTACTACTGGAGCTGGATCCG<br>CCAGTCCCCAGGGAAGGGGCTGGAGTGGATTGGGGAAATCAATGATAGTGGAAGCACCAACTACAACCCGTCCCTCAAGAGTCGA<br>GTCACCATATCAGTAGACACGTCCAAGAATCAGTTCTCCCTGAAACTGAGGTCTGTGACCGCCGCGGACACGGCTGTGTATTACT<br>GTGCGAGACCGTTCGACTATGACAGCCCGTTTGTGGAATGGGGCCAGGGAACCCTGGTCACCGTCTCCTCAG	97.98%
10S	IgK	GACATCCAGATGACCCAGTCTCCATCCTCCCTGTCTGCATCTGTAGGAGACAGAGTCACCATCACTTGCCGGGCAAGTCAGGGCA<br>TTAGAAATGATTTAGGCTGGTTTCAGCAAAAACCAGGGAAAGCCCCTAAGCGCCTCATCTATGCTGCATCCACTTTGCAAACTGG<br>GGCCCCATCGAGGTTCAGCGGCAGTGGATCTGGGACAGAATTCACTCTTACAATCACCAGCCTTCAGCCTGAAGATTTTGCAACT<br>TATTACTGTCTACAATATAATCATTACCCTCGGACGTTCGGCCAAGGGACCAAGGTGGAGATCAAAC	94.98%
11K	IgD,M	ATGAAACACCTGTGGTTCTTCCTCCTCCTGGTGGCAGCTCCCAGATGGGTCCTGTCCCAGGTGCAGCTGCAGGAGTCGGGCGCAG<br>GACTGTTGAAGCCTTCGGAGACCCTGTCCCTCACCTGCGCTGTCTATGGTGGGTCCTTCAGTGGTTACTACTGGAGCTGGATCCG<br>CCAGCCCCCAGGGAAGGGGCTGGAGTGGATTGGGGAAATCAATCATAGTGGAAGCACCAACTACAACCCGTCCCTCAAGAGTCGA<br>GTCACCATATCAGTAGACACGTCCAAGAACCAGTTCTCCCTGAAGCTGAGCTCTGTGACCGCCGCGGACACGGCTGTGTATTACT<br>GTGCGAGAGGAGGCTATGATAGTAGTGGTTATCCAAATGCTTTTGATATCTGGGGCCAAGGGACAATGGTCACCGTCTCTTCAG	98.95%
11K	IgL	CAGGCTGTGCTGACTCAGCCGTCTTCCCTCTCTGCATCTCCTGGAGCATCAGCCAGTCTCACCTGCACCTTACGCAGTGGCATCA<br>ATGTTGGTACCTACAGGATATACTGGTACCAGCAGAAGCCAGGGAGTCCTCCCCAGTATCTCCTGAGGTACAAATCAGACTCAGA<br>TAAGCAGCAGGGCTCTGGAGTCCCCAGCCGCTTCTCTGGATCCAAAGATGCTTCGGCCAATGCAGGGATTTTACTCATCTCTGGG<br>CTCCAGTCTGAGGATGAGGCTGACTATTACTGTATGATTTGGCACAGCAGCGTTTGGGTATTCGGCGGAGGGACCAAGCTGACCG<br>TCCTAG	100%
12O	IgM	ATGAAACACCTGTGGTTCTTCCTCCTCCTGGTGGCAGCTCCCAGATGGGTCCTGTCCCAGGTGCTTCTACAGCAGTGGGGCGCAG<br>GTCTGTTGAAGCCTTCGGAGACCTTGTCCCTCACTTGCGCTGTCTATGGTGAGTCCTTCAGTACTTCCCACTGGAGCTGGATCCG<br>CCAGCCCCCAGGGAAGGGGCTGGAGTGGATTGGCGACATAAATCATAGTGGAATCACCAAGTACAACCCGTCCCTCAAGAGTCGA<br>GTCACCATATCAATTGACACGTCCAAGAAGCAGTTTTCCCTGAAGCTGAGCTCCGTGACCGCCGCGGACACGGCTCTTTATTATT<br>GTGCGGGAAAGGGAAACATCTATGGCCCCCTTGACTACTGGGGCCAGGGAACCCTGGTCACCGTCTCCTCAG	91.93%
12O	IgK	GAAATTGTGTTGACACAGTCTCCAGGCACCCTGTCTTTGTCTCCAGGGGAAAGAGGCACCCTCTCCTGCAGGGCCAGTCAGAGTG<br>TTAACAGCACCTACTTAGCCTGGTACCAGCAAAAACCTGGCCAGGCTCCCAGGCTCCTCATCTCTGGTGCATCCACCAGGGCCAC<br>TGGCATCCCAGACAGGTTCAGTGGCAGTGGGTCTGGGACAGGCTTCACTCTCACCATCAGCAGACTGGAGCCTGAAGATTTTGCA<br>GTGTATTACTGTCAGCAGTGTGGTAATTCACCGTGCACTTTTGGCCAGGGGACCAAGCTGGACATCAAAC	96.1%
13S	IgG	ATGGACACACTTTGCTCCACGCTCCTGCTGCTGACCATCCCTTCATGGGTCTTGTCCCAGATTACCTTGAAGGAGTCTGGTCCTA<br>CGCTGGTGAAACCCACACAGACCCTCACACTGACCTGCACCTTGTCTGGGTTCTCGCTCACCACTCATGGAGTGGGTGTGGGCTG<br>GATCCGTCACCCCCCCGGAAAGGCCCTGGAATGGCTTGCGCTCATCTATTGGGATAATGATAAGCGCTACAGCCCCTCTCTGAAA<br>AAAAGACTCGCCATCGCCACAGACACGTCCAAAAAACAGGTGGTCCTTCGAATGACCGACATGGACCCTCTGGACACAGGCACAT<br>ATTATTGTGCCCACAGACACGTGGATCGGTACTTCGATGTCTGGGGCCGTGGCGCCCTAGTTTCTGTCTCCTCAG	89.96%
13S	IgK	GACATCGTGATGACCCAGTCTCCAGGCTCCCTGTCTTTGTCTCTGGGCGCGACGGCCACCATCACCTGCAAGGCCCAGTGGACTA<br>TCTTGTCCACCTCCAATAATAAGACTTACTTAGCCTGGTACCAAGCTAAACGAGGACGGCCTCCAAGATTACTCTTTTATTGGGC<br>GTCTAACCGACACTCCGGGGTCCCTGACCGATTCAGTGGCAGCGCTTCTGGAACGCATTTCGCTCTCACCATAAGGAACGTGCAG<br>GCTGAAGATGTGGCAGATTATTACTGTCAGCAACATAATAGTACTCCGTACACTTTTGGCCAGGGGACCAAGCTGGAGATCAAGC	82.49%
14K	IgM	ATGGAGTTTGGGCTGAGCTGGCTTTTTCTTGTGACTACTTTAAAAGGTGTCCGGTGTGAGGTGCAGTTATTGGAGTCTGGGGGAG<br>GCTTGGTTCAGCCGGGGGGTCCCTCAGACTCACCTGCGCAGCCTCTGGATTCACTTTTAATAATTATGCCATGAGTTGGGTCCG<br>CCAGTTTCCAGGGAAGGGGCTGGAGTGGGTCTCCACTATCAGTGGCACTGGTACTAGAACATTCTTCTCAGACTCCGTGACGGGC<br>CGCTTCACCATTTCTAGAGACAATGACAGGAACACGGTGTCTCTACAAATGAACTCCCTGAGAGTCGAGGACACGGCCGTCTATT<br>TCTGTGCGAAGAATTCGTCGCGGGACGTGGTATCTTCTGCTCTGGTCGGACAGAGCGGCGCTGAATATTTCCACCACTGGGGCCT<br>GGGCACTCTGGTCACTGTCTCCTCAG	85.76%
14K	IgK	GACATCGTGATGACCCAGTCTCCAGACTCCCTGGCTGTGTCTCTGGGCGAGAGGGCCACCATCAACTGCAAGTCCAGCCAGACTG<br>TTTTATACAACTCCAACAATAAAAACTACTTAGCTTGGTACCAGCAGAAACCAGGACAGCCTCCTAAGCTGCTCATTTACTGGGC<br>ATCTACCCGGGAATCCGGGGTCCCTGACCGTTTCAGTGGCAGCGGGTCTGGGACAGATTTCACTCTCACCATCAGCAGCCTGCAG<br>GCTGAAGATGTGGCAGTTTATTACTGTCAGCAATATTATAATACTCCGTACACTTTTGGCCAGGGGACCAAGCTGGAGATCAAAC	97.87%
15B	IgM	ATGGAGTTTGGGCTGAGCTGGGTTTTCCTTGTTACTATTATAAAAGGTGTCCAGTGTCAGGTGCAGCTGGTGGAGTCTGGGGGAG<br>GCTTGGTCAAGCCTGGAGGGTCCCTGAGACTCTCTTGTGTAGCCTCTGGATTCACCTTCAGTGACTACCATATGAGTTGGATCCG<br>CCAGGCTCCAGAGGGGGGGCTGGAGTGGCTTTCAAACATTAGTAGGAGTGGTGACACCATCAACTACGCAGACTCTGTCAAGGGC<br>CGATTTATCATCTCCAGGGACAACGCCAGGAATTCATTGTTTCTGCAAATGAACAGCCTGAGAGGCGAAGACACGGCCGTCTATT<br>ACTGTGCGAGGGGCGTTGGTGCGGTGGACTACTGGGGCCAGGGAACCCTGGTCACCGTCTCCTCAG	90.97%

15B	IgK	GACATCCAGATGACCCAGTCTCCATCCTCCCTGTCTGCATCTGTGGGAGACAGAGTCACCATCACTTGCCGGGCAAGTCACGACATTAGAAATGATTTAGGCTGGTATCAGCAGAAACCAGGGAAAGCCCCTAAGCGCCTAATCTACGCTGCATCTAGTTTGCAAAGTGGGGTCCCATCAAGGTTCAGCGGCAGTGGATCTGGGACAGAATTCACTCTCACAATCAGCAGCCTGCAGCCTGAAGATTTTGCAACTTATTTCTGTCTACAGCATGATAGTTATCCTCGCACTTTCGGCCAAGGGACACGACTGGACATTAACC	96.58%
16B	IgM	CAGGTGCAGCCACAACAGTGGGGCGCAGGACTGTTGAAGCCTTCGGAGACCCTGTCCCTCACCTGCGCTGTCTATGGTGGGTCCCTCAGTGGCTTCTTGTGGAGCTGGGTCCGCCAGCCCCCAGGGAAGGGGCTGGAGTGGATTGGAGAAATCAATTATAGTGGAAGTACCAACTACAATCCGTCCCTCAAGAGTCGAGTCACCATATCAGCAGACATGTCCAAGAACCAGTTCTCCCTGAAGTTGAACTCTGTGACCGCCGCGGACACGGCTGTTTACTACTGTGCGCCCGGCGGTTGGGGGGGTCTTGACTCCTGGGGCCAGGGAACCCTGGTCACCGTCTCCTCAG	94.04%
16B	IgK	GAAATTGTGTTGACGCAGTCTCCAGGCACCCTGTCTTTGTCTCCAGGGGAAAGAGCCACCCTCTCCTGCAGGGCCAGTCAGAGTGTTACTAACAGCTACTTAGCCTGGTACCAGCAGAAACCTGGCCAGGCTCCCAGGCTCCTCATCTATGGTGCATCCAGCAGGGCCACTGGCATCCCAGACAGGTTCAGTGGCAGTGGGTCTGGGACAGACTTCACTCTCACCATCAGCAGACTGGAGCCTGAAGATTTTGCAGTGTATTACTGTCAGCAATATGGTAGCCCACCTCGGACGTTCGGCCAAGGGACCAAGGTGGAAATCAAAC	98.58%
17K	IgM	GTGCAGCTGGTGCAGTCTGGACCAGAGGTGAAAAAGCCCGGGGAGTCTCTGAAGATCTCCTGTAAGGGTTCTGGATACAGCTTTACCACCTACTGGATCGGCTGGGTGCGCCAGATGCCCGGGAAAGGCCTGGAGTGGATGGGGATCATCTATCCTGGTGACTCTGATACCAGATACAGCCCGTCCTTCCAAGGCCAGGTCACCATCTCAGCCGACAAGTCCATCAGTACCGCCTACCTGCAGTGGAGCAGCCTGAAGGCCTCGGACAGCGCCATGTATTACTGTGCGAGACAGTCTGGGGGTCTTGACTACTGGGGCCAAGGGACCCTGGTCACCGTCTCCTCAG	98.60%
17K	IgK	GAGGTGCAGCTGGTGCAGTCTGGACCAGAGGTGAAAAAGCCCGGGGAGTCTCTGAAGATCTCCTGTAAGGGTTCTGGATACAGCTTTACCACCTACTGGATCGGCTGGGTGCGCCAGATGCCCGGGAAAGGCCTGGAGTGGATGGGGATCATCTATCCTGGTGACTCTGATACCAGATACAGCCCGTCCTTCCAAGGCCAGGTCACCATCTCAGCCGACAAGTCCATCAGTACCGCCTACCTGCAGTGGAGCAGCCTGAAGGCCTCGGACAGCGCCATGTATTACTGTGCGAGACAGTCTGGGGGTCTTGACTACTGGGGCCAGGGAACCCTGGTCACCGTCTCCTCA	98.61%
18B	IgM	GAGCTGGTTTCCTTGTGTTTTGTAGCAGGTGTCCAGTGTGAGGTGTATTTGGTGGAGTCTGGGGGAGGCTTGGTCCAGCCTGGGTCGTCCCTGAGACTCTCATGTGCAGCCTCTGGATTCATCTTTAATGACTTTTGGATGAGTTGGGTCCGCCAGGCTCCAGGGAAGGGACTGGAGTGGGTGGCCAATATAAATCAAGATGGACTTGAAAAGCATTATCTGGACTCTGTGAAGGGTCGATTCATCATATCCAGGGACAACACCAAGAGTTGTCTCTTTCTTCAAATGAACAACTTGAGAGTCGAGGACACGGCTAAATATTATTGCAGTGCCGGGGACTTCTTTGACTTCTGGGGCAAGGGAATCCTGGTCAGCGTCTCGTCAG	85.42%
18B	IgL	CAGTCTGTCGTGACGCAGCCGCCCTCAGTGTCTGGGACCCCCGGACAGACGGTCAGCATCTCTTGCTCTGGAAGCACTTCCAACATCGCAACTAATACCGTAAACTGGTACAGGCAAGTCCCAGGGACGGCCCCCAAACTCCTCATCTACACTAATGATCAGCGGCCCTCAGGAGTCACTGACCGATTCTCTGGCTCCAAGTCTGGCACCTCAGGATCCCTGGACATCAGTGGACTCCAGTCTGACGATGAGGCTGATTATTTTTGTGCAGCGTGGGATAGTGACCTGAGTGGATGGCTTTTCGGCGGAGGGACCAGGCTGACCGTCCTAG	87.02%
19V	IgG	ATGGAGTTTGGGCTGAGCTGGCTTTTTCTTGTGGCTAAAATAAAAGGTGTCCAGTGTGAGGTGCAGCTGTTGGAGTCTGGGGGAGGCTTGGTACAGCCTGGGGGGTCCCTGAGACTCTCATGTGGAGGCTCTGGATTCACCTTTAGTATGTTCGCCATGACCTGGGTCCGCCAGGCCCCGGGGAAGGGACTGGAGTGGGTCTCGACTATTTCTCGTGATGGAGCGTCCACATCATACGCAGACTCCGTGAAGGGCCGGTTCACCATCTCCAGAGACAACGCCAAGAACACCTTATGTCTGCAAATGAACAGCCTGAGAGTCGAAGACACGGCCGTCTATTATTGTACGAAGTCGCCGTATTCCACCAGTTGCTTGCGGTCCCCTCAATATTTCCACCGTTGGGGCCAGGGCACCCTGGTCAACGTCTTCTCAG	87.50%
19V	IgL	TCCTATGTGCTGACTCAGCCACCCTCGGTGTCAGTGACCCCAGGAAAGACGGCCAAGATTCCCTGTGGGGGACATAAACTTGGAGGTGAAAGTGTCCACTGGTACCAGCAGATGCCAGGCCAGGCCCCTGTGTTGGTCATCCATGATGACACTGAGCGGCCCTCAGGGATCCCAGACCGATTCTCTGGCTCCAAGTCTGGGAGCACGGCCACCCTGACCATCAGCACGGTCGAGGCCGGGGATGAGGCCGACTATTTTTGTCAGGTGTGGAATACTACTCGTGATCATGCGGTGTTCGGCGGAGGGACCAAGCTGACCGTCCTTG	89.61%

Variable gene segment (VH, Vk, VL) are green, joining (J) are blue, non-template or N(D)N—red.

Table S2. Idiotype peptides for patients 7T and 12O, used in study.

Peptide	Sequence
7T_VH_39	VSCKSSGDTFNKYAL
7T _VH_62	QGPEWMGSIIPLLGT
7T _VH_97	TTYLELKSLRFEDTA
7T _VH_117	RGWSGTGTASGFNYW
7T _Vk_186	ASQSVSANSLAWYQQ
7T _Vk_198	YQQKPGRPPRLLIY
7T _Vk_207	RLLIYGAFSRATGIP
7T _Vk_248	YYCQHYGSSPRTFGQ
12O_VH_20	QVLLQQWGAGLLKPSETL
12O_VH_42	AVYGESFSTSHWSW
12O_VH_66	WIGDINHSGITKYNP
12O_VH_85	RVTISIDTSKKQFSL
12O_VH_113	YCAGKGNIYGPLDY
12O_VH_120	IYGPLDYWGQGTLVT
12O_Vk_46	RLLISGASTRATGI
12O_Vk_86	VYYCQQCGNSPCTFGQ

Germline VH/Vk sequence is green, JH/Jk is blue, non-template—red.
